# The consumption of psychoactive substances among French physicians: how do they perceive the creation of a dedicated healthcare system?

**DOI:** 10.3389/fpsyt.2023.1249434

**Published:** 2023-12-14

**Authors:** Bénédicte Jullian, Marine Deltour, Nicolas Franchitto

**Affiliations:** ^1^Centre Hospitalier de Toulouse-Purpan, Service d’Addictologie Universitaire, Toulouse, France; ^2^CERPOP, Université de Toulouse, Inserm, UPS, Toulouse, France

**Keywords:** French physicians, alcohol related disorders, audit, care for physicians, dedicated healthcare system

## Abstract

**Introduction:**

Substance use among physicians can have negative impacts on their health, quality of life, and patient care. While Physician Health Programs (PHPs) have proven effective, many physicians with substance use disorders (SUDs) still face obstacles in seeking help. Our study explores the expectations, attitudes, and experiences of French physicians regarding the implementation of a specialized healthcare system (SHS) for addiction, and their opinions on the factors that could improve the effectiveness of such a service, with a focus on substance use disorders (SUDs).

**Methods:**

We conducted a web-based survey from April 15 to July 15, 2021, which included questions about sociodemographic characteristics, substance use, and attitudes toward a specialized healthcare system (SHS) for physicians with SUDs.

**Results:**

Of the 1,093 respondents (62.5% female), 921 consumed alcohol (84.2%), and 336 (36.4%) were categorized as hazardous drinkers (AUDIT-C ≥ 4 for women and ≥ 5 for men). The mean AUDIT-C score was 3.5 (±1.7 SD), with a range from 1 to 12. Factors associated with hazardous alcohol consumption included coffee consumption [OR 1.53 (1.11–2.12)], psychotropic drug use [OR 1.61 (1.14–2.26)], cannabis use [OR 2.96 (1.58–5.55)], and other drug use [OR 5.25 (1.92–14.35)]. On the other hand, having children was associated with non-hazardous alcohol consumption [OR 0.62 (0.46–0.83)]. Only 27 physicians (2.9%) had consulted a specialist in addiction medicine, while 520 (56.4%) expressed interest in such a consultation. The main barriers to accessing a dedicated consultation were denial (16.3%), physician self-medication (14.3%), fear of judgment (12.8%), and confidentiality concerns (10.2%).

**Conclusion:**

A specialized consultation with trained professionals in a neutral location can improve access to care for healthcare workers and maintain patient confidentiality and anonymity. Prevention and awareness can reduce addiction stigma and help peers in need. The improvement of healthcare workers’ addiction culture and detection of addictive behavior in peers depends on academic addiction medicine.

## 1 Introduction

Substance use disorders (SUDs) are prevalent among physicians worldwide, with potential consequences for patient outcomes and safety. According to American numbers, the lifetime prevalence of SUD in physicians is slightly higher (15.4%) than in the general population (12.6%) (1). In Europe, hazardous alcohol and drug use among physicians were estimated at 18–23 and 3%, respectively (2–5). In France, data regarding French physicians’ alcohol behaviors are scarce, but hazardous drinking and consumption of hypnotics seem to increase especially among young physicians (6). Globally, physicians often seek treatment for substance use disorders (SUDs) at a later stage, which can negatively impact their own health and patient care (7–9). Substance use treatment and harm reduction services are crucial for maintaining the well-being of physicians. Physician health programs (PHPs) have been established in various countries, such as the United States, Canada, Australia, United Kingdom, Spain, Norway, and Switzerland, to help doctors with addiction recover through abstinence-based programs and adherence monitoring (10–13). In France, regular monitoring through random workplace visits and screenings does not exist. Although recent studies conducted during the COVID-19 pandemic have shown negative changes in SUD among physicians in France, there is no information available on the type of treatment and monitoring offered (6, 14, 15).

Our aim is to explore the expectations, attitudes, and experiences of French physicians regarding the implementation of a specialized healthcare system (SHS) for addiction, and their opinions on the factors that could improve the effectiveness of such a service, with a focus on substance use disorders (SUDs).

## 2 Methods

### 2.1 Population and data collection

Between April 15 and July 15, 2021, any French physician was invited to participate in the survey without restriction criteria provided they could complete the questionnaire autonomously by clicking on a URL link. The link leading to the online survey was disseminated on the internet using social media (i.e., Twitter, LinkedIn, and Facebook) and national media. The recruitment strategy thus followed a convenience sampling method. No registration was needed to participate in the survey and no individually identifying details were collected from participants, which is compliant with the French general data protection regulation and local laws (16). Approval for this study was obtained from the local institutional review board at the University of Toulouse (number RnIPH 2021–141). The survey was designed to be completed in less than 15 min. Participants completed the survey voluntarily, with the knowledge that the data would be anonymized that they were free to stop at any point during their completion of the survey, and that incomplete surveys were discarded (17). To prevent individuals from completing the questionnaire multiple times, only one questionnaire could be submitted from a particular IP address. Specialties were categorized into groups according to their similarities as found elsewhere (18): general practice, intensive care (anesthesia, intensive care), emergency, medicine (internal medicine, hepato-gastro-enterology or cardiology for example) and surgery (orthopedic or otorhinolaryngology for example).

### 2.2 Measures

The data were collected via a survey that was divided into three parts.

The first part collects sociodemographic characteristics (gender, age, current living situation, professional situation, a number of years of practice). The second part gathers information about substance use. We chose the Alcohol Use Disorders Identification Test, version C (AUDIT-C) to assess drinking habits in this anonymous survey because of its brevity and clarity (19, 20). The French version of the AUDIT has been validated (21) and the details in AUDIT-C scoring can be found elsewhere (22). A score of 5 points or more was used as an indicator of hazardous drinking (23), which is defined as a pattern of alcohol consumption that increases the risk of harmful consequences for the user or others. A score ≥ 5 for men and ≥ 4 for women be the most consistent and corresponds to the optimal operating point in terms of sensitivity and specificity (≥ 5 points for men: sensitivity 91.2% and specificity 95.2%, and ≥ 4 points for women: sensitivity 81.4% and specificity 93.1%) (1, 19, 21, 23, 24). Answers to the third question of the AUDIT-C were also used to categorize binge drinking (any mention of 6 drinks or more per occasion), because similar data was available for the general population. Distinction between hazardous consumption and dependence could not be made, because the three items of the AUDIT-C reflect only alcohol consumption (23, 25).

We assessed health behaviors, including tobacco and cannabis use, and coffee and psychotropic consumption. Tobacco dependence was evaluated on the basis of the time of the first cigarette (smoking within 30 mn of waking up) and the number of cigarettes smoked per day. Cannabis use was assessed by asking about frequency in the past year, while coffee consumption was measured by self-reported daily intake. Psychotropic consumption was assessed by asking “are you under psychotropic medication?”

In the third section of our survey, we inquired about the potential implementation of a specialized healthcare system (SHS) for physicians struggling with SUD. Participants were asked to share factors that hinder or facilitate the delivery of health services and were assessed by asking: “Why do you think healthcare professionals with addiction issues rarely seek help from the nearest addiction treatment service? I have no idea/Fear of breaching medical confidentiality with the administration/Healthcare professionals self-treated/There is no need to seek treatment until there is a problem/Fear of job loss or demotion/Limited knowledge about the organization of addiction treatment follow-up/Fear of being stigmatized/Fear of being judged because of their profession as a healthcare professional.

Additionally, we asked about their level of interest and expectations regarding the implementation of a dedicated consultation, which were rated on a scale from 1 (very interested) to 6 (less interested).

### 2.3 Statistical analysis

Statistical analyses were conducted using Stata Version 16.0 software (StataCorp LP, College Station, TX). For the comparison of qualitative variables, we used either the Pearson’s chi-square test or Fisher’s exact test as appropriate. Problematic alcohol consumption was compared to non-harmful alcohol consumption with a univariate analysis, and then with a multivariate analysis (variables which were statistically significant on univariate analysis at *p* < 0.20 were included in the model). Therefore, the multivariate analysis was a stepwise downward logistic regression. *p* value <0.05 was considered significant.

## 3 Results

One thousand and ninety-three physicians completed the survey. Among them, 921 admitted alcohol consumption (84.2%) and 172 were abstinent (15.7%). Table 1 summarizes the participants’ sociodemographic and occupational characteristics. The majority of respondents were aged 26 to 45 years old (63.3%), and 62.5% were women. 278 worked in intensive care units (30.1%), a quarter was general practitioners (26.6%), and the almost 30% of them were medical specialists. The majority has been working for more than 5 years (71.6%). Almost 10 % of them are current smokers: they are long-time smokers (mean length of smoking: 17.3 years), smoking manufactured cigarettes for 87.9% of them, and smoking the first cigarette over 1 h after waking-up for the majority (59.7%). Fifty-nine admitted cannabis consumption (6.4%) over the last year, but mostly less than once per month (59.3%). 70.7% consumed coffee, with a mean consumption of 3 cups per day. Two hundred and one (21.8%) were under psychotropic medications (benzodiazepines, hypnotics and anti-depressants). Finally, 3% reported consuming other substances such as cocaine (41.9%) or MDMA (67.7%) every month.

**Table 1:** Sociodemographic and occupational characteristics of physicians.

N	921
Age, n (%)	
26–35 (years-old)	318 (34.5%)
36–45 (years-old)	266 (28.8%)
46–55 (years-old)	175 (19.0%)
56–65 (years-old)	145 (15.7%)
>65 (years-old)	17 (1.8%)
Gender, n (%)	
Male	345 (37.4%)
Female	576 (62.5%)
Specialties, n (%)	
Specialist in Addiction medicine/psychiatry	127 (13.7%)
Surgical specialties	45 (4.8%)
Intensive care	278 (30.1%)
General practice	245 (26.6%)
Emergency department	42 (4.5%)
Medical specialties	184 (19.9%)
Length of practice, n (%)	
<2 years	80 (8.7%)
2–5 years	180 (19.6%)
>5 years	658 (71.6%)
Professional status, n (%)	
University hospital physicians	289 (31.8%)
Hospital physicians	242 (26.2%)
CSAPA^1^/CARUUD^2^	27 (2.9%)
Private practice	221 (24.0%)
Private clinic	145 (15.7%)
Family situation, n (%)	
Single	149 (17.2%)
In a relationship	715 (82.7%)
Childless	243 (37.3%)
With children	576 (62.6%)
Tobacco	
Daily consumption	
No	829 (90.0%)
Yes	92 (9.9%)
Duration (years)	
N (%)	91 (98.9%)
Mean (SD)	17.3 (10.5)
Number of cigarettes per day	
N (%)	92 (100.0%)
0–10	64 (69.5%)
11–20	23 (25.0%)
21–30	2 (2.1%)
31 and over	3 (3.2%)
Delay between wake-up and first cigarette	
N (%)	92 (100.0%)
<5 min	3 (3.2%)
6–30 min	17 (18.4%)
31–60 min	17 (18.4%)
Over 1 h	55 (59.7%)
Cannabis	
Consumption	
No	862 (93.5%)
Yes	59 (6.4%)
Over the last year	
N (%)	59 (100.0%)
<1 time/month	35 (59.3%)
1 time/month	4 (6.7%)
2/3 times/month	5 (8.4%)
1 time/week	6 (10.1%)
>4 times/week	9 (15.2%)
Coffee	
No	269 (29.2%)
Yes	652 (70.7%)
Number of cups per day?	
N (%)	646 (99.0%)
Mean (SD)	3.2 (2.0)
Psychotropic medications	
No	720 (78.1%)
Yes	201 (21.8%)
Type	
Benzodiazepines	123 (61.1%)
Hypnotics	63 (31.3%)
Anti-depressants	42 (20.8%)
Others	33(16.3%)
Other substances	
No	890 (96.6%)
Yes	31 (3.3%)
Type	
Cocaine	13 (41.9%)
MDMA	21 (67.7%)
Miscellaneous	10 (32.2%)

^1^CSAPA: Health support and addiction-prevention centers. ^2^CAARUD: Care and risk-reductions centers.

Table 2 summarizes the results of the AUDIT-C score. The mean AUDIT-C score was 3.5 (±1.7 SD) and the extreme ranged from 1 to 12. Three hundred and thirty-six physicians (36.4%) were included in the group “hazardous drinking” (AUDIT-C ≥ 4 for women and ≥ 5 for men). Physicians aged between 26 and 35 years (40.4%) and males (63.1%) were prevalent, with a sex ratio of 1.7. They have been working for at least 2 years (23.5%), are single (21.4%) with no child (46.5%) compared to those with no risky alcohol consumption [respectively 17.3% (*p* < 0.01), 14.7% (*p* < 0.05) and 32.0% (*p* < 0.001)]. During the past year, 474 (51.4%) physicians reported at least one event involving more than 6 drinks.

**Table 2:** Factors associated with hazardous alcohol consumption.

AUDIT score	All	Univariate analysis			Multivariate analysis
		Non-harmful alcohol consumption	Hazardous drinking	*p*	Odds Ratio [IC95%]
N (%)	921 (100.0%)	585 (63.5%)	336 (36.4%)	*–*	
Age, n (%)					
26–35 (years-old)	318 (34.5%)	182 (31.1%)	136 (40.4%)	*<0.05*	
36–45 (years-old)	266 (28.8%)	170 (29.0%)	96 (28.5%)		
46–55 (years-old)	175 (19.0%)	122 (20.8%)	53 (15.7%)		
56–65 (years-old)	145 (15.7%)	100 (17.0%)	45 (13.3%)		
>65 (years-old)	17 (1.8%)	11 (1.8%)	6 (1.7%)		
Gender, n (%)					
Male	345 (37.4%)	221 (37.7%)	124 (36.9%)	*NS*	–
Female	576 (62.5%)	364 (62.2%)	212 (63.1%)		
Specialties, n (%)					
Addictology/psychiatry	127 (13.7%)	85 (14.5%)	42 (12.5%)	*NS*	–
Surgical specialties	45 (4.8%)	25 (4.2%)	20 (5.9%)		
Intensive care	278 (30.1%)	168 (28.7%)	110 (32.7%)		
General practice	245 (26.6%)	159 (27.1%)	86 (25.6%)		
Emergency department	42 (4.5%)	30 (5.1%)	12 (3.5%)		
Medical specialties	184 (19.9%)	118 (20.1%)	66 (19.6%)		
Length of practice, n (%)					
<2 years	80 (8.7%)	43 (7.3%)	37 (11.0%)	*<0.01*	–
2–5 years	180 (19.6%)	101 (17.3%)	79 (23.5%)		
>5 years	658 (71.6%)	439 (75.3%)	219 (65.3%)		
Professional status, n (%)					
University hospital physicians	289 (31.8%)	171 (29.2%)	118 (35.1%)	*NS*	–
Hospital physicians	242 (26.2%)	164 (28.0%)	78 (23.2%)	*NS*	
CSAPA^1^/CARUUD^2^	27 (2.9%)	19 (3.2%)	8 (2.3%)	*NS*	
Liberal cabinet	221 (24.0%)	146 (24.9%)	75 (22.3%)	*NS*	
Private clinic	145 (15.7%)	88 (15.0%)	57 (16.9%)	*NS*	
Family situation, n (%)					
Single	149 (17.2%)	80 (14.7%)	69 (21.4%)	*<0.05*	–
In a relationship	715 (82.7%)	462 (85.2%)	253 (78.5%)		
Childless	243 (37.3%)	187 (32.0%)	156 (46.5%)	*<0.001*	–
With children	576 (62.6%)	397 (67.9%)	179 (53.4%)		
During past year, at least one event involving more than 6 drinks?					
Never	447 (48.5%)	415 (70.9%)	32 (9.5%)	*<0.001*	1
<1 time/month	314 (34.0%)	163 (27.8%)	92 (27.3%)		12.26 [7.90–19.02]
1 time/month	98 (10.6%)	6 (1.0%)	92 (27.3%)		221.03 [83.17–587.41]
1 time/week	48 (5.2%)	1 (0.1%)	47 (13.9%)		566.11 [75.30–4255.93]
Almost every day	14 (1.5%)	0	14 (4.1%)		1
Tobacco, n (%)					
No	829 (90.0%)	536 (91.6%)	293 (87.2%)	*<0.05*	–
Yes	92 (9.9%)	49 (8.3%)	43 (12.8%)		
Cannabis, n (%)					
N	862 (93.5%)	566 (96.7%)	296 (88.1%)	*<0.001*	–
Yes	59 (6.4%)	19 (3.2%)	40 (11.9%)		
Coffee, n (%)					
No	269 (29.2%)	191 (32.6%)	78 (23.2%)	*<0.005*	–
Yes	652 (70.7%)	394 (67.3%)	258 (76.7%)		
Psychotropics, n (%)					
No	720 (78.1%)	480 (82.0%)	240 (71.4%)	*<0.001*	1
Yes	201 (21.8%)	105 (17.9%)	96 (28.5%)		1.97 [1.23–3.14]
Other substances					
No	890 (96.6%)	580 (99.1%)	310 (92.2%)	*<0.001*	–
Yes	31 (3.3%)	5 (0.8%)	26 (7.7%)		

NS: non-significant. ^1^CSAPA: Health support and addiction-prevention centers. ^2^CAARUD: Care and risk-reductions centers.

Other consumption habits were more often reported such as tobacco (12.8% vs. 8.3%, p < 0.05), cannabis (11.9% vs. 3.2%, *p* < 0.001), coffee (76.7% vs. 67.3%, *p* < 0.005), psychotropic drugs (28.5% vs. 17.9%, p < 0.001) and cocaine (7.7% vs. 0.8%, *p* < 0.01). There was no significant difference according to gender or medical specialty.

In the multivariate analysis, the factors independently and significantly associated with hazardous alcohol consumption were coffee consumption [OR 1.53 (1.11–2.12)], being treated with psychotropic drugs [OR 1.61 (1.14–2.26)], smoking cannabis [OR 2.96 (1.58–5.55)] and other drug use [OR 5.25 (1.92–14.35)]. Conversely, having children was associated with non-hazardous alcohol consumption [OR 0.62 (0.46–0.83)].

Physicians were asked whether they had already sought help, whatever the SUD (Table 3). Twenty-seven physicians (2.9%) reported a previous specialized consultation more often a specialist in addiction medicine (48.1%). Among the surveyed physicians, 520 (56.4%) expressed interest in visiting a specialist in addiction medicine if such a SHS was available. Various barriers to accessing dedicated consultations were identified, including denial (16.3%), physicians self-medicating (14.3%), fear of being judged (12.8%), and concerns about confidentiality (10.2%). Around 66.9% of physicians placed high importance on privacy and anonymity when seeking resources for substance use disorder (SUD) care. A neutral setting and consultations with trained caregivers were also considered significant by 20–27% of participants. On the other hand, flexible time scheduling was perceived as the least important aspect (Table 4).

**Table 3:** Interest and barriers in visiting a specialized healthcare system in addiction medicine.

	N (%)
Have you ever visited a specialist in addiction medicine given your consumption habits?	
No	894 (97.0%)
Yes	27 (2.9%)
Specialist in addiction medicine	13 (48.1%)
Psychiatrist	10 (37.0%)
General practitioner	2 (7.4%)
Psychologist	6 (22.2%)
Other	2 (7.4%)
Do you think it would be helpful for you to visit a specialist in addiction medicine?	
No	827 (89.7%)
Yes	94 (10.2%)
If there was a dedicated healthcare system for addiction medicine, would you consider visiting a specialist in that field?	
No	401 (43.5%)
Yes	520 (56.4%)
In your opinion, what are the barriers to accessing a specialized healthcare system in addiction medicine?	
No idea	8 (0.8%)
Fear of breach of medical secrecy	94 (10.2%)
Caregivers treat themselves	132 (14.3%)
No need to consult as long as no problem	27 (2.9%)
Fear of job loss/downgrading	26 (2.8%)
Lack of knowledge in the organization of addiction monitoring	33 (3.5%)
Fear of being stigmatized	80 (8.6%)
Fear of being judged	118 (12.8%)
Denial	151 (16.3%)

**Table 4:** Physicians’ views on the establishment of a specialized healthcare system (SHS) in addiction medicine, from the most interesting (1) to the least interesting (6).

	n	Mean rating* (SD)	1	2	3	4	5	6
Privacy/anonymity	911	2.1 (1.8)	66.9%	7.2%	4.2%	2.6%	3.8%	15.0%
Neutral setting	907	2.9 (1.7)	26.7%	23.1%	13.8%	10.0%	12.4%	13.6%
Service independence	908	3.2 (1.8)	24.0%	16.9%	16.6%	12.4%	11.4%	18.5%
Care providers who have received training in providing care for physicians	907	3.2 (1.6)	20.8%	17.7%	18.0%	15.3%	14.8%	13.1%
Flexible time scheduling	909	3.4 (1.7)	18.4%	15.2%	18.7%	13.8%	13.5%	20.1%

*Mean rating calculated only among responses for individuals who responded to the question.

## 4 Discussion

This survey shows that 36.4% of physicians have hazardous alcohol consumption, with illegal substance consumption more frequently associated. We did not show any difference between the different medical specialties as previously described in France (18), although some studies showed that anesthesiologists are overrepresented (26). Focusing on the third question of AUDIT – C, our results are slightly higher compared with other studies where binge drinking ranged between 13.5–19.5% of European physicians (2, 4, 27). As previously studied, we observed a lower smoking rate among health care professionals compared with other professionals (28). The number of physicians taking psychotropic drug (28.5% in our study) is not surprising as a significant part of SUD diagnoses among physicians is related to their authority to prescribe drugs and their easier access to prescription drugs (29). The prevalence of illicit drug use varies among studies and is difficult to describe. In France, in 2005, a national study involving anesthetists found a prevalence of 2.6% of cannabis users (30). These differences in the prevalence of SUD, especially with illicit substances, echo the lack of quality data worldwide regarding the prevalence of substances most probably misused by professionals in different regions throughout the world.

Given our specific interest in help-seeking for SUD among physicians, it was important to obtain a snapshot of their substance consumption habits, in order to align with their views on the establishment of a specialized healthcare system (SHS) in addiction medicine. Currently, PHPs in multiple countries offer resources for physicians with SUD, providing assessment, monitoring, coordination of formal treatment, and regular workplace visits and screenings, but not treatment itself (10). Nonetheless, the outcomes of PHPs have shown to be significantly more positive than outcomes for individuals with SUD in the general population, and they have resulted in the best long-term outcomes for individuals with SUDs. Because the goal is to address physicians suffering from SUDs, it is mandatory that the response of SHS be adapted in order to promote an alliance in care and the patient-physician’s adherence to the proposed contract. This fluidity of care requires that the specialized team in SUD adapts and overcomes the access barriers to care described in the literature to address addiction-related issues among their members and employees (13, 31–33). In line with previous studies (34–36), the more frequent barriers cited in our study included denial of the SUD, fear of being judged, or experiencing stigma. This statement suggests that certain lead to delays in receiving necessary care and can exacerbate health conditions as physicians neglect their own health by self-diagnosing and self-prescribing medications (37). Furthermore, the statement suggests that physicians may also underestimate or ignore their own impairment, further hindering their ability to receive the care they need, reminding that the situation has not improved and the barriers remain unchanged when compared to surveys conducted a few years ago (38).

For the surveyed physicians, the location of the SUD treatment program in a neutral place is important, which relates to the concept of respecting confidentiality. For physicians working within a healthcare facility, it is understandable that if hospitalization is necessary for SUD or the treatment of an associated psychiatric or somatic comorbidity, it should be done in a separate facility. For outpatient care of physician-patients, confidentiality can be further improved if consultations occur outside the addiction medicine service in a building dedicated to physicians and, by extension, all healthcare professionals. This statement refers to the barriers of the healthcare system itself. This modern concept of separating the barriers to accessing healthcare not only relies on the patient-doctor relationship, but also on a change in the hospital itself, and should be encouraged (35, 36, 39). The reorganization or creation of dedicated spaces for healthcare providers within the healthcare facility is still to be accepted. Although healthcare providers should receive the same quality of care as the general population, it is not possible to ignore that treating physicians is a unique situation and that a responsive system may be a solution to facilitate adherence to follow-up care.

Respect of confidentiality emphasizes the delicate balance that physicians who are treating these “physicians-patients” are facing between the preservation of medical confidentiality and the obligation to report physicians who are unable to deliver competent medical services because of their SUDs (13, 40). However, more and more, participation is becoming a confidential and voluntary option, serving as an alternative to discipline sanctions. Another difficulty inherent in treating physicians with SUD is that their care can only be part of a comprehensive approach, including a complete history that covers psychiatric, medical, family, social, legal, educational, and occupational factors. Occupational health physicians play a crucial role in the care of physician-patients by leveraging their understanding of issues encountered by health workers (36). In this regard, their role is essential in proactively identifying and addressing risks for patients, physicians in recovery, and the workplace. Moreover, during addiction treatment, the process of returning to work should be carefully planned and monitored, with a gradual, staged approach to incorporating occupational activities that carry more risk. Occupational health physicians play a key role in adjusting work schedules and facilitating the medical care organization through part-time work (36, 41). However, in France, the Code of Medical Deontology stipulates that medical confidentiality is not shared between the treating physician, i.e., the addiction medicine specialist, and the occupational health physician. Therefore, it is mandatory that the physician-patient be informed and that the appointment with the occupational health physician be initiated by the physicians -patients themselves.

### 4.1 Implementing changes

If a specialized addiction program is available, physicians are more likely to seek assistance from addiction teams, indicating that addressing addiction issues may be a more effective starting point for care than addressing psychiatric comorbidities that may discourage impaired physicians from seeking help (8, 9, 36). We recently introduced a program called Addictions Confidential Consultations for Help and Care for Healthcare Workers (ACCESS) to meet this requirement. The program’s interdisciplinary team comprises addiction specialists, psychiatrists, psychologists, social workers, nurses, and a general practitioner, addressing the absence of a single point of care for physicians (39). The program provides holistic biopsychosocial care for physicians struggling with SUDs. The first consultation is conducted by a trained psychiatrist-addiction specialist, with experience in treating caregivers, an essential aspect of building a strong therapeutic alliance. Given the frequent association of psychiatric comorbidities with SUD, they are an essential element of the comprehensive evaluation process. Caregivers are trained in providing care to other caregivers through a university training program,^1^ as highlighted by physicians who took part in the research, strengthening confidence in the specialized team.

### 4.2 Limits

This study should be interpreted in light of several limitations. Although the response rate for the survey was acceptable, young physicians were overrepresented in our study, as they may be more Internet-affine than their older colleagues. Therefore, this bias is not specific to this survey. Distinction between hazardous consumption and dependence could not be made, because the three items of the AUDIT-C reflect only alcohol consumption (23, 25). Second, specialties were grouped partly based on convenience. Because of the broad sample of specialties with small numbers per specialty we were unable to perform analyses at the level of individual specialties. Thirdly, the study design, which relied on self-reported consumption, and the lack of information about clinical and other psychosocial variables that could potentially influence responses regarding the criteria for implementing a program for physician-patients with SUD.

## 5 Conclusion

A specialized consultation with trained professionals in a neutral location, respecting patient confidentiality and anonymity, could improve access to care for healthcare workers. Professionals trained in addiction medicine could develop a coordinated and contractualized care journey. Awareness and prevention actions can reduce negative representations of addiction and support peers in difficulty. This approach is part of the teaching mission of academic addiction medicine to improve the addiction culture of healthcare workers and identify addictive behavior in their peers.

## Data availability statement

The raw data supporting the conclusions of this article will be made available by the authors, without undue reservation.

## Ethics statement

No registration was needed to participate in the survey and no individually identifying details were collected from participants, which is compliant with the French general data protection regulation and local laws. Approval for this study was obtained from the local institutional review board at the University of Toulouse (number RnIPH 2021–141).

## Author contributions

BJ and NF designed the study. MD built up and updated the database. NF and MD were involved in data synthesis, data interpretation. All authors provided critical feedback, contributed to the article, and approved the submitted version.

## Footnotes

^1^ http://diu-soignerlessoignants.fr/

## References

[1] Oreskovich MR Shanafelt T Dyrbye LN Tan L Sotile W Satele D. The prevalence of substance use disorders in American physicians. Am J Addict. (2015) 24:30–8. doi: 10.1111/ajad.12173 25823633

[2] Rosta J. Hazardous alcohol use among hospital doctors in Germany. Alcohol Alcohol. (2008) 43:198–203. doi: 10.1093/alcalc/agm180, PMID: 18208862

[3] Rosta J Aasland OG. Female surgeons’ alcohol use: a study of a national sample of Norwegian doctors. Alcohol Alcohol. (2005) 40:436–40. doi: 10.1093/alcalc/agh186 16043434

[4] Joos L Glazemakers I Dom G. Alcohol use and hazardous drinking among medical specialists. Eur Addict Res. (2013) 19:89–97. doi: 10.1159/000341993, PMID: 23128570

[5] Sorensen JK Pedersen AF Vedsted P Bruun NH Christensen B. Substance use disorders among Danish physicians: an explorative study of the professional socialization and management of colleagues with substance use disorders. J Addict Med. (2016) 10:248–54. doi: 10.1097/ADM.0000000000000228 27379820

[6] Fond G Boulangeat C Messiaen M Duba A Boucekine M Auquier P. Anxiety and depression in young physicians: prevalence and associated factors. MESSIAEN national study Encephale. (2022) 48:26–30. doi: 10.1016/j.encep.2021.02.005, PMID: 33892920

[7] Geuijen P de Rond M Kuppens J Atsma F Schene A de Haan H. Physicians’ norms and attitudes towards substance use in colleague physicians: a cross-sectional survey in the Netherlands. PloS One. (2020) 15:e0231084. doi: 10.1371/journal.pone.0231084, PMID: 32243472 PMC7122818

[8] Khong E Sim MG Hulse G. The identification and management of the drug impaired doctor. Aust Fam Physician. (2002) 31:1097–100. PMID: 12516511

[9] Rosen A Wilson A Randal P Pethebridge A Codyre D Barton D. Psychiatrically impaired medical practitioners: better care to reduce harm and life impact, with special reference to impaired psychiatrists. Australas Psychiatry. (2009) 17:11–8. doi: 10.1080/10398560802579526, PMID: 19137466

[10] McLellan AT Skipper GS Campbell M DuPont RL. Five-year outcomes in a cohort study of physicians treated for substance use disorders in the United States. BMJ. (2008) 337:a2038–6. doi: 10.1136/bmj.a2038 18984632 PMC2590904

[11] Hegenbarth C. Rescuing doctors in distress. CMAJ. (2011) 183:E153–4. doi: 10.1503/cmaj.109-3760 21262938 PMC3042466

[12] Rø KEI Gude T Aasland OG. Does a self-referral counselling program reach doctors in need of help? A comparison with the general Norwegian doctor workforce. BMC Public Health. (2007) 7:36. doi: 10.1186/1471-2458-7-36 17367526 PMC1847681

[13] Braquehais MD Valero S Bel MJ Navarro MC Matali JL Nasillo V. Doctors admitted to a physicians’ health program: a comparison of self-referrals versus directed referrals. BMJ Open. (2014) 4:e005248. doi: 10.1136/bmjopen-2014-005248 PMC4091506 24993767

[14] Frajerman A Colle R Hozer F Deflesselle E Rotenberg S Chappell K. Psychological distress among outpatient physicians in private practice linked to COVID-19 and related mental health during the second lockdown. J Psychiatr Res. (2022) 151:50–6. doi: 10.1016/j.jpsychires.2022.04.003, PMID: 35447507 PMC9002100

[15] Rolland F Hadouiri N Haas-Jordache A Gouy E Mathieu L Goulard A. Mental health and working conditions among French medical students: a nationwide study. J Affect Disord. (2022) 306:124–30. doi: 10.1016/j.jad.2022.03.001 35276314 PMC8902864

[16] Lemaire F. The Jardé law: what does change. Presse Med. (2019) 48:238–42. doi: 10.1016/j.lpm.2019.01.006 30853280

[17] Greenberg N Weston D Hall C Caulfield T Williamson V Fong K. Mental health of staff working in intensive care during Covid-19. Occup Med. (2021) 71:62–7. doi: 10.1093/occmed/kqaa220 PMC7928568 33434920

[18] Thiebaud PC Martin C Naouri D le Joncour A Truchot J Yordanov Y. Alcohol consumption among French physicians: a cross-sectional study. Drug Alcohol Depend. (2021) 218:108356. doi: 10.1016/j.drugalcdep.2020.108356 33342514

[19] Reinert DF Allen JP. The alcohol use disorders identification test: an update of research findings. Alcohol Clin Exp Res. (2007) 31:185–99. doi: 10.1111/j.1530-0277.2006.00295.x, PMID: 17250609

[20] Bush K Kivlahan DR McDonell MB Fihn SD Bradley KA. The AUDIT alcohol consumption questions (AUDIT-C): an effective brief screening test for problem drinking. Ambulatory Care Quality Improvement Project (ACQUIP). Alcohol Use Disorders Identification Test. Arch Intern Med. (1998) 158:1789–95. 9738608 10.1001/archinte.158.16.1789

[21] Gache P Michaud P Landry U Accietto C Arfaoui S Wenger O. The alcohol use disorders identification test (AUDIT) as a screening tool for excessive drinking in primary care: reliability and validity of a French version. Alcohol Clin Exp Res. (2005) 29:2001–7. doi: 10.1097/01.alc.0000187034.58955.64, PMID: 16340457

[22] Mo D Min K Gluck R Jiang F Tao R Geng F. Alcohol use and misuse among Chinese psychiatrists during the early COVID-19 pandemic. Front Psych. (2022) 13:933814. doi: 10.3389/fpsyt.2022.933814 PMC9258332 35815044

[23] Rumpf HJ Hapke U Meyer C John U. Screening for alcohol use disorders and at-risk drinking in the general population: psychometric performance of three questionnaires. Alcohol Alcohol. (2002) 37:261–8. doi: 10.1093/alcalc/37.3.261 12003915

[24] Sebo P Bouvier Gallacchi M Goehring C Künzi B Bovier PA. Use of tobacco and alcohol by Swiss primary care physicians: a cross-sectional survey. BMC Public Health. (2007) 7:5. doi: 10.1186/1471-2458-7-5, PMID: 17222332 PMC1781430

[25] Dawson DA Grant BF Stinson FS Zhou Y. Effectiveness of the derived alcohol use disorders identification test (AUDIT-C) in screening for alcohol use disorders and risk drinking in the US general population. Alcohol Clin Exp Res. (2005) 29:844–54. doi: 10.1097/01.ALC.0000164374.32229.A2, PMID: 15897730

[26] Lefebvre LG Kaufmann IM. The identification and management of substance use disorders in anesthesiologists. Can J Anaesth. (2017) 64:211–8. doi: 10.1007/s12630-016-0775-y 27900672

[27] Romero-Rodríguez E Pérula De Torres LÁ Fernández García JÁ Parras Rejano JM Roldán Villalobos A Camarelles GF. Alcohol consumption in Spanish primary health care providers: a national, cross-sectional study. BMJ Open. (2019) 9:e024211. doi: 10.1136/bmjopen-2018-024211, PMID: 30782898 PMC6377550

[28] O’Keeffe A Hayes B Prihodova L. “Do as we say, not as we do?” the lifestyle behaviours of hospital doctors working in Ireland: a national cross-sectional study. BMC Public Health. (2019) 19:179. doi: 10.1186/s12889-019-6451-8 30744600 PMC6371571

[29] Geuijen P Schellekens A Schene A Atsma F. Substance use disorder and alcohol consumption patterns among Dutch physicians: a nationwide register-based study. Addict Sci Clin Pract. (2023) 18:4. doi: 10.1186/s13722-022-00356-9, PMID: 36639645 PMC9837897

[30] Beaujouan L Czernichow S Pourriat JL Bonnet F. Prevalence and risk factors for substance abuse and dependence among anaesthetists: a national survey. Ann Fr Anesth Reanim. (2005) 24:471–9. doi: 10.1016/j.annfar.2005.02.023, PMID: 15904727

[31] DuPont RL McLellan AT Carr G Gendel M Skipper GE. How are addicted physicians treated? A national survey of physician health programs. J Subst Abuse Treat. (2009) 37:1–7. doi: 10.1016/j.jsat.2009.03.010 19482236

[32] DuPont RL McLellan AT White WL Merlo LJ Gold MS. Setting the standard for recovery: physicians’ health programs. J Subst Abuse Treat. (2009) 36:159–71. doi: 10.1016/j.jsat.2008.01.004 19161896

[33] Merlo LJ Greene WM. Physician views regarding substance use-related participation in a state physician health program. Am J Addict. (2010) 19:529–33. doi: 10.1111/j.1521-0391.2010.00088.x, PMID: 20958849 PMC2959195

[34] Kunyk D Inness M Reisdorfer E Morris H Chambers T. Help seeking by health professionals for addiction: a mixed studies review. Int J Nurs Stud. (2016) 60:200–15. doi: 10.1016/j.ijnurstu.2016.05.001 27297381

[35] Geuijen PM Pars E Kuppens JM Schene AH De Haan HA De Jong CAJ. Barriers and facilitators to seek help for substance use disorder among Dutch physicians: a qualitative study. Eur Addict Res. (2022) 28:23–32. doi: 10.1159/000517043 34192705

[36] Vayr F Herin F Jullian B Soulat JMJM Franchitto N. Barriers to seeking help for physicians with substance use disorder: a review. Drug Alcohol Depend. (2019) 199:116–21. doi: 10.1016/j.drugalcdep.2019.04.004, PMID: 31035230

[37] Samuelson ST Bryson EO. The impaired anesthesiologist: what you should know about substance abuse. Can J Anaesth. (2017) 64:219–35. doi: 10.1007/s12630-016-0780-1, PMID: 27928715

[38] Benkhadra K Adusumalli J Rajjo T Hagen PT Wang Z Murad MH. A survey of health care needs of physicians. BMC Health Serv Res. (2016) 16:472. doi: 10.1186/s12913-016-1728-4, PMID: 27600560 PMC5013614

[39] Kay M Mitchell G Clavarino A Doust J. Doctors as patients: a systematic review of doctors’ health access and the barriers they experience. Br J Gen Pract. (2008) 58:501–8. doi: 10.3399/bjgp08X319486, PMID: 18611318 PMC2441513

[40] Bismark MM Morris JM Clarke C. Mandatory reporting of impaired medical practitioners: protecting patients, supporting practitioners. Intern Med J. (2014) 44:1165–9. doi: 10.1111/imj.12613, PMID: 25442757 PMC4313682

[41] Cossin T Thaon I Lalanne L. Workaholism prevention in occupational medicine: a systematic review. Int J Environ Res Public Health. (2021) 18:7109. doi: 10.3390/ijerph18137109, PMID: 34281048 PMC8297306

